# Clinical Management of Localized Aggressive Periodontitis With Esthetic Replacement of Tooth 11 Using a Resin-Bonded Bridge: A Case Report

**DOI:** 10.7759/cureus.102312

**Published:** 2026-01-26

**Authors:** Sara K Abulfateh, Shahad M Albinali, Bushra F Althawadi, Shaikha E Al-Doseri

**Affiliations:** 1 Department of Dentistry, Royal Medical Services, Bahrain Defense Force, Manama, BHR; 2 Department of Periodontology, Royal Medical Services, Bahrain Defense Force, Manama, BHR

**Keywords:** aggressive periodontitis, localized periodontitis, molar-incisor pattern, nonsurgical periodontal therapy, resin bonded bridge, tooth loss management

## Abstract

Aggressive periodontitis (AgP) is a severe periodontal disease that causes early tooth loss in healthy young patients. Localized aggressive periodontitis (LAP) often affects specific teeth with rapid destruction of supporting tissues, causing functional and esthetic problems. Conservative treatment options are needed to manage tooth loss and maintain oral health.

This case report describes the case of LAP in a 33-year-old man who presented with clinical attachment loss and bone destruction affecting the molars and incisors. Clinical and radiographic examination revealed deep periodontal pockets, angular bone defects, and mobility of tooth 11. A diagnosis of localized periodontitis was made. The patient underwent nonsurgical periodontal therapy (NSPT) with adjunctive antibiotics. Despite treatment, tooth 11 had a poor prognosis and was extracted. An immediate denture was fabricated, and a resin-bonded bridge (RBB) was made to restore esthetics and function. The patient was stable and was closely monitored with regular periodontal maintenance.

This case report presents a comprehensive management of a patient with AgP, highlighting the importance of early diagnosis and addressing the challenges of AgP. Conservative approaches such as NSPT, RBBs, and splinting strategies helped to maintain function and improve oral health.

## Introduction

Aggressive periodontitis (AgP) is a rapidly progressing form of periodontal disease characterized by early onset and significant attachment loss in otherwise healthy individuals [1]. A localized form of AgP, known as localized aggressive periodontitis (LAP), typically affects younger individuals with minimal plaque deposits [2]. LAP involves rapid destruction of the supporting tissues, specifically affecting the first molar and central incisor teeth, which can lead to tooth loss. A common clinical manifestation of AgP is deep periodontal pockets and attachment loss that are seen as a molar-incisor pattern [3]. In addition, gingival recession is observed, commonly affecting the labial surfaces of anterior teeth [2]. In such cases, this poses a restorative challenge, particularly when considering the age of the patient and the need for conservative treatment options [4].

A primary periodontal lesion originates from plaque-induced periodontal disease, affecting the supporting structures of the tooth. Over time, deep periodontal pockets may extend apically to communicate with the pulp through accessory canals or the apical foramen, leading to secondary pulpal involvement. Clinically, the tooth may show deep pockets, gradual bone loss, and later loss of vitality. The initial treatment is periodontal therapy; endodontic treatment is only required if pulpal necrosis occurs [5].

The classification of periodontal diseases was updated in the 2017 World Workshop on the Classification of Periodontal and Peri-Implant Diseases and Conditions, which introduced a transformative update to the understanding and diagnosis of periodontal health and disease. This comprehensive classification system, developed collaboratively by the American Academy of Periodontology and the European Federation of Periodontology, replaced the 1999 Armitage classification to reflect decades of advances in research and clinical practice [6]. The new 2017 classification of periodontal diseases identifies three main forms of periodontitis: necrotizing periodontitis; periodontitis (combining former chronic and aggressive types), staged I-IV and graded A-C; and periodontitis as a manifestation of systemic disease. Grading encompasses three grades: grade A (minimal risk for progression), grade B (moderate risk for progression), and grade C (high risk for progression) [6]. It also classifies periodontal health and gingivitis (plaque-induced and non-plaque-induced), mucogingival conditions, traumatic occlusal forces, and factors related to teeth or prostheses. For implants, it defines peri-implant health, peri-implant mucositis, peri-implantitis, and hard- and soft-tissue deficiencies, providing a comprehensive framework for diagnosis and management [6].

The management of AgP presents unique challenges due to its rapid progression and the risk of tooth loss [7]. Given that periodontitis is among the most common oral diseases, its impact extends beyond oral structures, influencing overall health and well-being. Nonsurgical periodontal therapy (NSPT) is the foundational approach for managing periodontal diseases, aiming to control infection and prevent disease progression [7]. It plays a vital role in stabilizing the periodontal condition and setting the stage for successful restorative interventions. In addition, systemic antibiotic therapy (a combination of amoxicillin and metronidazole) is usually prescribed as an adjunct to NSPT to eliminate pathogenic bacteria and improve clinical outcomes [1,3].

In younger patients with tooth loss due to LAP, restorative treatment should maintain function and esthetics while preserving natural tooth structure. Resin-bonded bridges (RBBs), also known as resin-retained or adhesive bridges, are one of the replacement options for missing teeth, requiring little to no preparation of adjacent teeth, making them suitable for patients with high aesthetic demands and largely unrestored dentitions [4]. Among the available designs, metal-framed RBBs (especially cantilever types) have shown reliable performance and long-term success [4]. In addition, RBBs and splinting techniques help stabilize mobile teeth, restore occlusal harmony, improve patient comfort, and contribute to the longevity of the dentition by distributing occlusal forces more evenly [8].

## Case presentation

**Figure 1:**
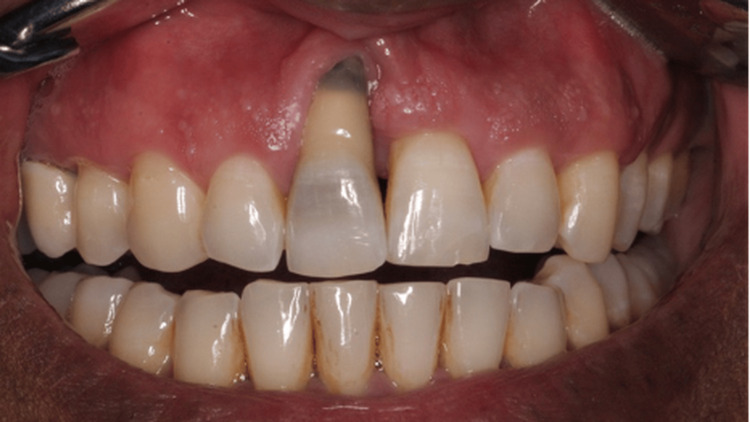
Intraoral photograph showing tooth 11 with severe gingival recession

A 33-year-old male patient with no known allergies or medical alerts presented to the dental clinic with a primary complaint of gum recession (Figure 1). He has asthma, which is under control, and he identifies as an ex-smoker, having quit over a decade ago. The patient presented with a chief complaint of progressively increasing gingival recession and swelling, which had begun to affect his comfort and ability to maintain oral hygiene. He reported a history of fixed orthodontic treatment that lasted three years. He generally maintained good oral hygiene but had irregular dental follow-up visits. He denied any associated systemic symptoms such as fever or malaise. The patient was concerned about the esthetic appearance and requested assessment and treatment.

Extraoral examination showed nothing abnormal. Intraoral examination revealed good oral hygiene. Soft-tissue examination revealed a significant soft-tissue loss of the interdental papilla and clinical attachment loss of the upper right central incisor gingiva. The patient's Basic Periodontal Examination was scored 4 in the upper right and upper middle sextants, indicating pockets larger than 5.5 mm in these quadrants. The lower middle quadrant scored 3, indicating that the patient has pockets ranging from 3.5 to 5.5 mm in this quadrant. The patient has fixed orthodontic retainers in place from teeth (14-24 and 44-34) following previous orthodontic treatment. A cold test with ethyl chloride was performed on tooth 11 and showed a normal positive response. Tooth 11 was tender to percussion. Mobility was found in teeth 16, 15, 11, 21, and 36.

**Figure 2:**
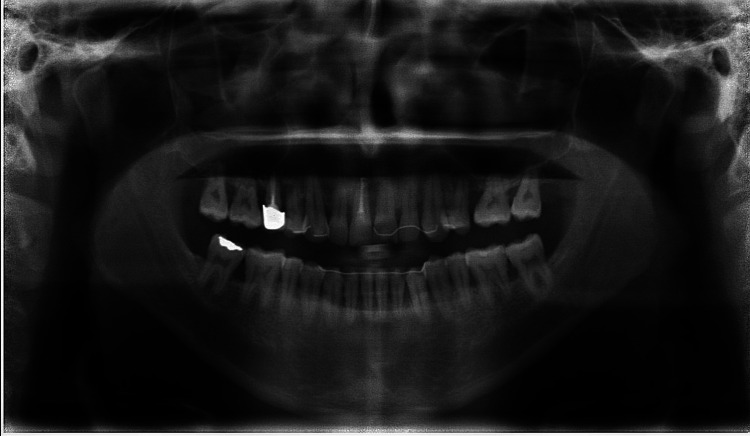
DPT taken during the treatment course, following completion of the root canal therapy on tooth 11, demonstrates generalized moderate bone loss DPT: dental panoramic tomography

A dental panoramic tomography was taken during the treatment course, following completion of the root canal therapy on tooth 11, revealing generalized moderate bone loss, with severe bone loss around teeth 17, 16, and 11 (Figure 2).

Based on the clinical and radiographic examination, the following differential diagnoses were made: 1) localized periodontitis, 2) periodontal abscess, and 3) primary periodontal-secondary endodontic lesion. The diagnosis of Stage IV, Grade C periodontitis in this case was made following the 2017 World Workshop criteria. As defined in the classification system, staging is based on severity and complexity, including clinical attachment loss, percentage of radiographic bone loss, probing depth (10 mm mesial to tooth 11), angular defects, furcation involvement, and mobility due to periodontitis [6]. There is severe gingival recession on tooth 11 and multiple pockets greater than 5.5 mm. Moreover, tooth mobility affects the function. Stage IV is diagnosed when the severity of destruction combined with functional complications requires comprehensive multidisciplinary rehabilitation [6].

The patient was informed of the condition, including the potential outcomes and treatment options. Prognosis was deemed poor for teeth 11 and 16. Oral hygiene instructions (OHIs) were provided, and periodontal indices were recorded. NSPT was initiated, including root surface debridement (RSD) under local anesthesia using 2% lidocaine with 1:100,000 adrenaline. Antibiotics were prescribed (amoxicillin and metronidazole) as an adjunct to NSPT. Follow-up visits showed improved plaque control and soft-tissue stability. OHIs were continuously reinforced, and interdental cleaning was emphasized using TePe brushes (TePe Munhygienprodukter AB, Malmö, Sweden), with the appropriate brush size as required.

**Figure 3:**
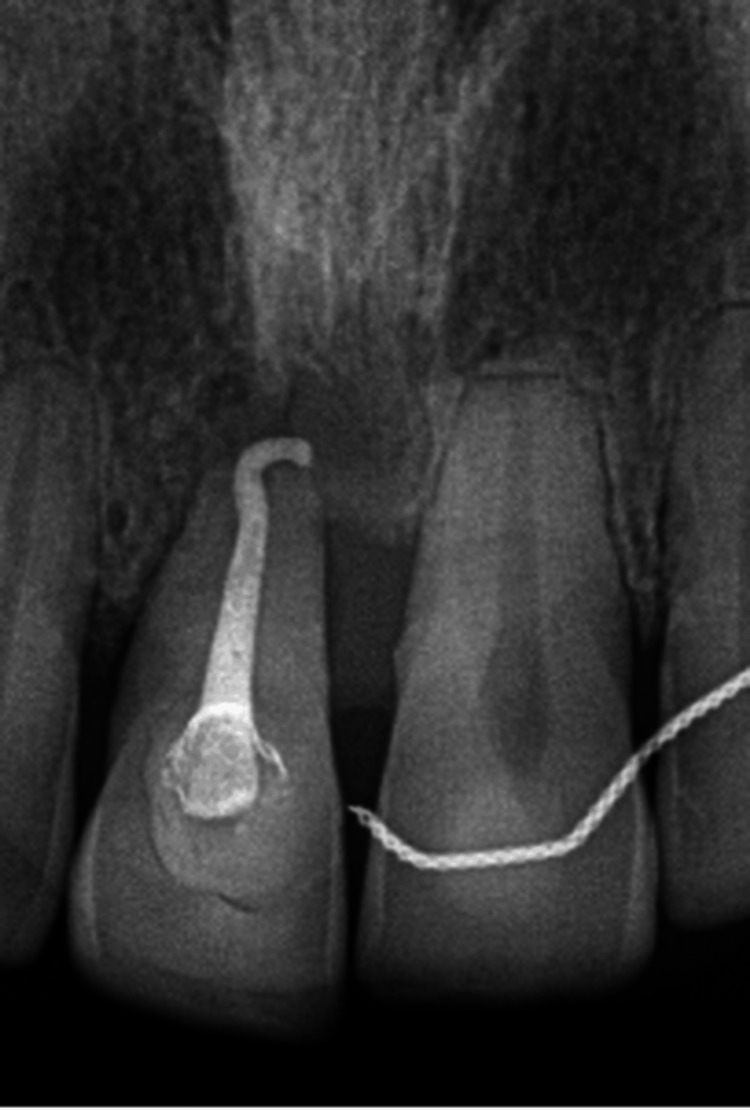
Intraoral periapical radiograph showing root canal treatment of tooth 11 with apical external root resorption

**Figure 4:**
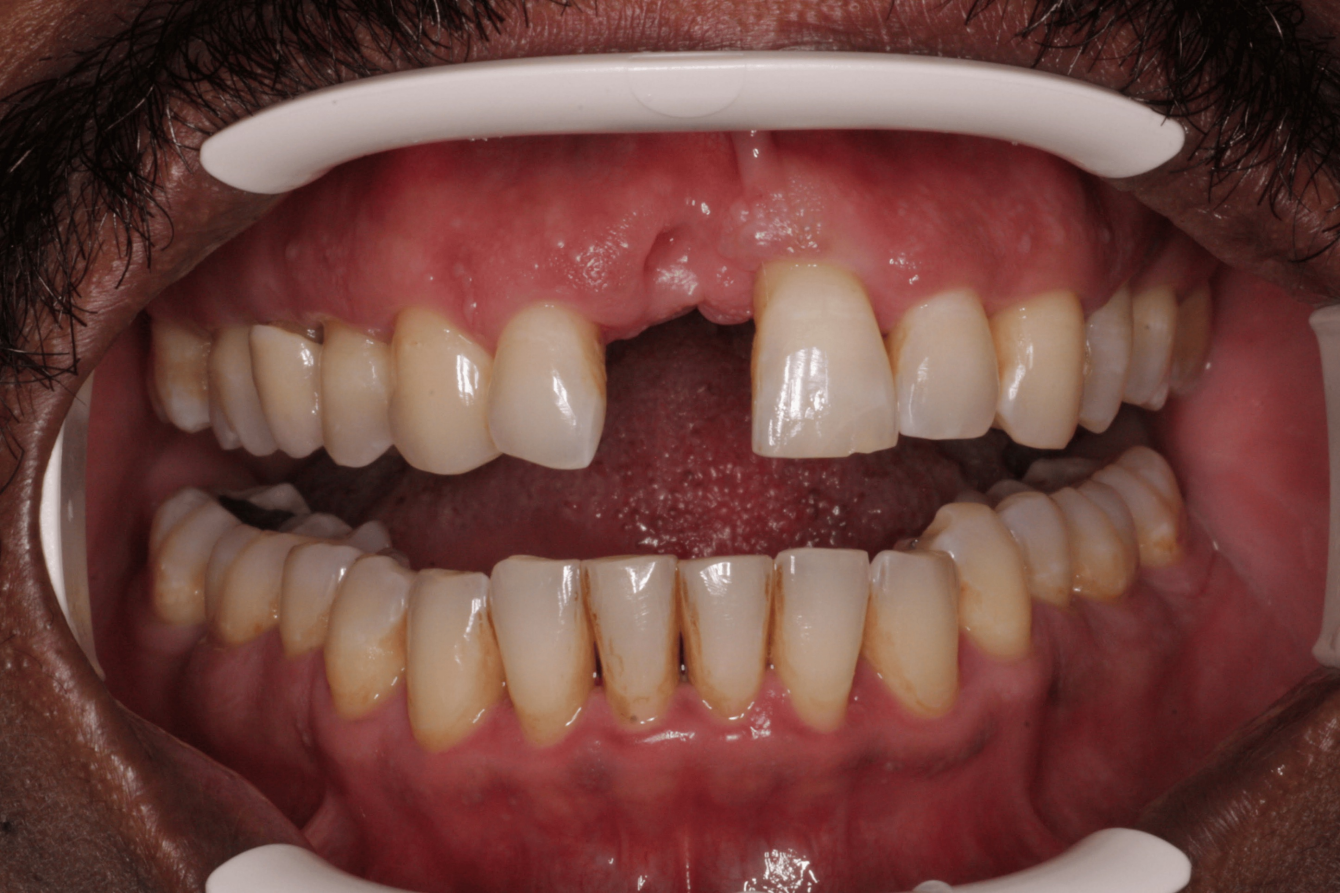
Intraoral photograph showing a healed socket after extraction of tooth 11

**Figure 5:**
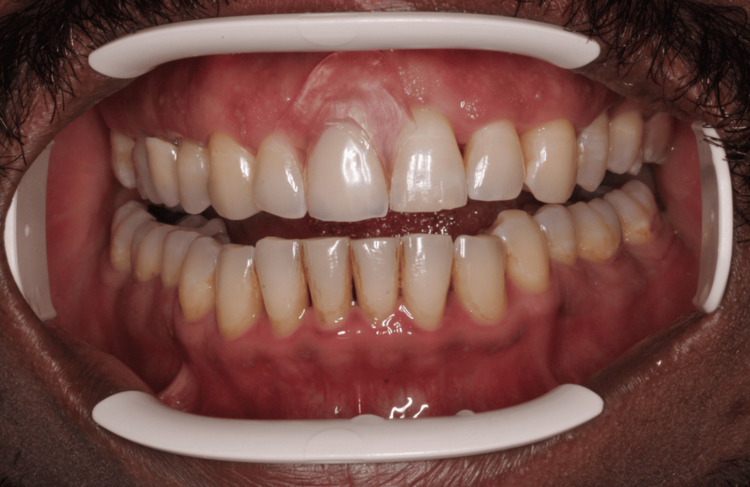
Intraoral photograph showing immediate denture

During follow-up visits, clinical examination revealed a papule in the labial vestibule of tooth 11 with a positive response to the electric pulp test. This confirmed that the lesion was periodontal in origin and not endodontic. The patient was referred to an endodontist to initiate root canal treatment (RCT) on tooth 11 to rule out possible endodontic involvement. The upper fixed retainer was cut to facilitate access prior to RCT. However, after assessment and an RCT, root resorption was noted on radiographic examination, leading to the decision to extract tooth 11 (Figure 3). Impressions were taken, and an immediate denture was fabricated before extraction. The patient was informed of the potential soft-tissue defect that might compromise esthetics. The extraction was performed, and the immediate denture was delivered. At the follow-up appointment, an intraoral photograph demonstrated a healed socket with no signs of bleeding or inflammation, and a photograph of the patient wearing the immediate denture was also taken (Figures 4, 5).

**Figure 6:**
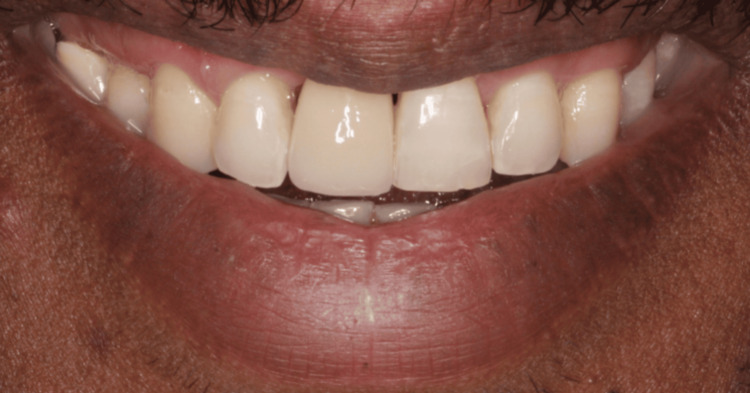
Smile view

A definitive replacement option, RBB, was later planned and performed to replace tooth 11, extending from tooth 13 to 23 for splinting and to improve functional and esthetic outcomes. The patient was satisfied with the result and was advised to continue maintenance and to have a review (Figure 6).

Throughout the management, the patient's oral hygiene was closely monitored and reinforced. Adjustments to interdental cleaning aids were made, and proper technique was regularly demonstrated. Regular periodontal maintenance and follow-up appointments were advised to support long-term stability.

## Discussion

LAP, a relatively uncommon but severe form of periodontitis, is characterized by fast attachment loss and alveolar bone degradation [6]. First molars and central incisors are frequently affected [6]. Early diagnosis and treatment are crucial because, in comparison to chronic periodontitis, LAP develops in young individuals who are otherwise systemically healthy [3]. It poses treatment challenges because it spreads quickly and exhibits an unusual pattern, often requiring a combined treatment approach [1].

Even though periodontal diseases are usually treated by dentists and periodontists, early detection depends on general healthcare professionals, parents, and even teachers being aware of the condition [7]. Since LAP usually starts around adolescence and can cause severe tooth loss if left untreated, early detection is essential [6]. Unfortunately, patients frequently come with significant periodontal damage because of a lack of regular check-ups and public awareness [1,2].

To better characterize the severity and progression risk of the disease, Caton et al. redefined periodontitis and introduced staging and grading [6]. This method places LAP under the category of molar/incisor pattern periodontitis, highlighting its aggressive yet limited features [3]. Prognosis and treatment plan modification depend on precise staging and grading of the disease [9].

In this case, NSPT, which includes scaling, root planing, and strict reinforcement of oral hygiene, is the basis of controlling LAP [7]. Amoxicillin and metronidazole are examples of additional systemic antibiotics that have shown advantages in reducing microbial load and slowing the progression of disease, according to Midwood and Hodge [7]. Long-term success still depends on patients' compliance with maintenance and dental hygiene.

Even with careful treatment, LAP can lead to early tooth loss, especially in the esthetic anterior region, which may result in functional and psychological issues [2]. Definitive implant therapy may also be further delayed due to costs [3,10,11]. RBBs are a reasonable immediate solution in these situations [4,12], as they are used in this case as a replacement option for missing teeth.

RBBs provide minimally invasive ways to restore function and appearance without requiring major surgery or tooth preparation [4,13]. Rathee et al. used both conventional and digital processes to demonstrate their usefulness for replacing lost teeth in the esthetic zone [12]. Advantages of RBBs include esthetic enhancement, preservation of the residual tooth structure, and relatively low cost [12]. However, because periodontal stability is important to success, they need to be carefully chosen [4,13].

Temporary splinting using fiber reinforcement or stainless steel wire mixed with composite resin may help secure teeth and distribute occlusal forces in cases of residual mobility brought on by inadequate periodontal support [8]. Sekhar et al. demonstrated the effectiveness of fiber splints in enhancing patient comfort and function, showing results similar to those with conventional wire-composite splints in cases of chronic periodontitis [8]. As in the case, splinting was done using the RBB itself.

Proper care, including scaling/root planing and clear OHIs to manage plaque, is very important for long-term control of LAP, as this was done for this patient. Regular check-ups are done for the detection of the recurrence of the disease [4,12,14].

A thorough, diverse approach that prioritizes early detection, patient education, and strict maintenance is required due to the aggressive nature and early onset of LAP. When tooth loss occurs, conservative temporary approaches like RBBs offer beneficial functional and esthetic rehabilitation until patients are ready for more permanent procedures like implants. To maximize results, individualized treatment programs that consider patient preferences, growth status, periodontal stability, and financial considerations are crucial [1-4,6-8,12,13].

Compared with typical presentations of LAP reported in the literature, such as the cases by Malhotra [1] and Dash et al. [2] involving young patients, often in their teens or early 20s, with rapid attachment loss around first molars and incisors, our patient’s case stands out due to his age and more localized presentation. At 33 years old, with a history of generally good oral hygiene following orthodontic treatment and no significant systemic symptoms, this patient differs from the classical LAP profile described by Kim et al. [3] and Caton et al. [6]. Nevertheless, he presented with progressively enlarging gingival swelling that compromised both esthetics and plaque control, an important trigger for periodontal diseases. Given his stable systemic health, well-controlled asthma, and long-standing cessation of smoking, nonsurgical management strategies recommended by Midwood and Hodge were done, which include RSD OHIs and adjunct antibiotics [7]. Additionally, considering the localized tooth mobility and patient concerns about appearance, we used an RBB both as a splinting mechanism and as a conservative esthetic replacement, consistent with approaches supported by Miettinen and Millar [4], King et al. [13], and Rathee et al. [12]. Unlike some cases where immediate implants or more invasive procedures are deferred due to age-related growth considerations, in our patient’s case, the RBB served as a minimally invasive, cost-effective, and patient-pleasing solution that complemented the ongoing periodontal therapy, emphasizing the tailored, multifactorial decision-making required in managing periodontal cases.

## Conclusions

This case highlights the successful management of LAP in an adult patient using a conservative, nonsurgical approach. Early diagnosis, combined with NSPT, antibiotics, and careful follow-up, helped stabilize the condition. Although tooth 11 had to be extracted due to poor prognosis, the use of an RBB provided a simple and esthetic solution that maintained both function and appearance. Regular maintenance and patient cooperation played a key role in the outcome. This case shows how personalized treatment plans and minimally invasive options can effectively manage aggressive periodontal disease and improve the patient’s oral health and quality of life.
